# Left bundle branch area pacing from the iliac approach in a patient without superior access: a case report

**DOI:** 10.1093/ehjcr/ytae486

**Published:** 2025-02-11

**Authors:** Lei Xu, Yangang Su, Shengmei Qin, Junbo Ge

**Affiliations:** Department of Cardiology, Zhongshan Hospital, Fudan University, 180 Fenglin Road, Shanghai 200032, China; National Clinical Research Centre for Interventional Medicine, 180 Fenglin Road, Shanghai 200032, China; Department of Cardiology, Zhongshan Hospital, Fudan University, 180 Fenglin Road, Shanghai 200032, China; National Clinical Research Centre for Interventional Medicine, 180 Fenglin Road, Shanghai 200032, China; Department of Cardiology, Zhongshan Hospital, Fudan University, 180 Fenglin Road, Shanghai 200032, China; National Clinical Research Centre for Interventional Medicine, 180 Fenglin Road, Shanghai 200032, China; Department of Cardiology, Zhongshan Hospital, Fudan University, 180 Fenglin Road, Shanghai 200032, China; National Clinical Research Centre for Interventional Medicine, 180 Fenglin Road, Shanghai 200032, China

**Keywords:** Left bundle branch area pacing, Iliac vein, Case report, Bilateral brachiocephalic vein occlusion

## Abstract

**Background:**

Left bundle branch area pacing (LBBAP) emerges as an alternative to traditional right ventricular pacing, demonstrating safety and feasibility through superior access for implantation.

**Case summary:**

An 89-year-old female, with a prior history of pacemaker pocket infection and a tricuspid endocarditis during follow-up, was referred to our hospital for management due to a sinus arrest. The patient underwent pacemaker removal due to a pocket infection in the right subclavian area, necessitating tricuspid valvuloplasty for infective endocarditis. Venography revealed occlusion in the left brachiocephalic vein. Subsequently, LBBAP pacemaker implantation via the right iliac vein was performed, with follow-up indicating proper functioning of the pacing system.

**Discussion:**

The feasibility of the right iliac vein access for LBBAP implantation is highlighted, demonstrating good stability and offering a practical alternative. This approach mitigates unnecessary risks associated with thoracotomy and epicardial lead placement, providing a safer and effective option for cardiac pacing.

**Learning points**

- The feasibility of iliac vein access for left bundle branch area pacing implantation is alternative when the superior venous access is not feasible.
- Compared with the femoral vein, the advantage of iliac access is that the leads are unaffected by lower limb activities.

## Introduction

Traditional right ventricular apex pacing (RVAP) presently stands as the conventional method, boasting simplicity, low dislocation risk, and high safety. However, being a non-physiological pacing mode, RVAP may induce ventricular electrical and mechanical dyssynchronization, elevating the chances of heart failure and atrial fibrillation.^1^ While His–Purkinje system pacing is the ideal physiological pacing mode, its high threshold and challenging implantation limit widespread adoption.^2,3^ In recent years, Huang *et al.*^4^ introduced left bundle branch area pacing (LBBAP), demonstrating effective achievement of ventricular electrical and mechanical synchrony. Numerous studies have affirmed the safety and feasibility of LBBAP through superior access.^5–7^ In this report, we detail a case of pacemaker implantation using LBBAP through the iliac approach.

## Summary figure

**Figure:**
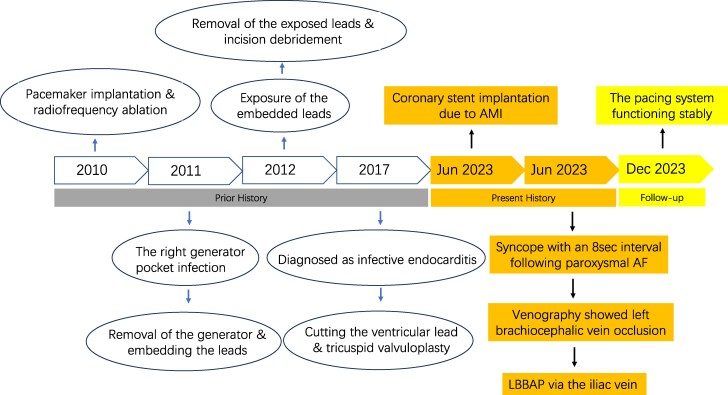


## Case summary

**Figure 1:**
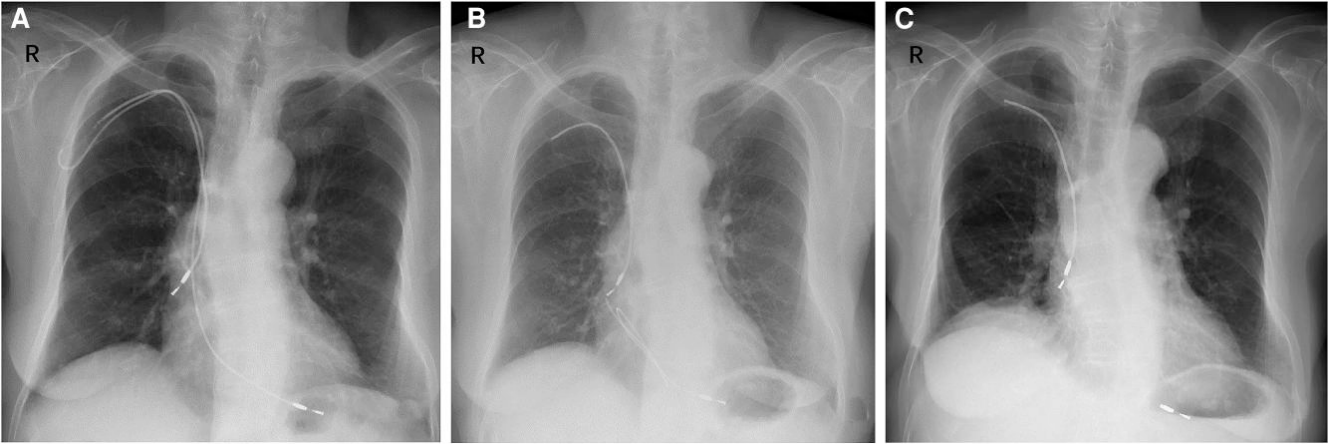
(*A*) The X-ray after the first debridement (the generator was removed, and the leads were embedded subcutaneously). (*B*) The X-ray after the second debridement (the exposed lead on the right subclavian area was cut off). (*C*) The X-ray after cutting the ventricular lead, removing the bacterial neoplasm under the tricuspid valve and performing the tricuspid valvuloplasty.

An 89-year-old Chinese female, presenting with recurrent syncope due to pre-automatic pause, was admitted to our hospital. In 2010, she underwent pacemaker implantation and atrial fibrillation radiofrequency ablation in another facility, utilizing right subclavian vein access for right atrial and ventricular lead placement. Subsequently, a pocket infection occurred, leading to the removal of the generator and subcutaneous embedding of the leads (*Figure 1A*). Given the patient’s maintained sinus rhythm, no further pacemaker implantation was performed. However, subsequent exposure of the embedded leads at the right subclavian area prompted hospital admission for lead removal and incision debridement (*Figure 1B*). In April 2017, the patient experienced fever and chills, resulting in an infective endocarditis diagnosis. Echocardiography indicated a potential neoplasm on the right ventricular pacemaker lead, along with mild pulmonary hypertension and moderate tricuspid regurgitation. The germ of the blood culture showed that *Corynebacterium* was positive. The cardiac surgeon conducted surgery, involving further cutting of the ventricular lead, removal of the bacterial neoplasm beneath the tricuspid valve, and tricuspid valvuloplasty (*Figure 1C*). In June 2023, the patient underwent percutaneous coronary stent implantation due to acute anterior wall myocardial infarction. Subsequently, she suffered syncope with an 8-s-long pre-automatic pause, leading to her transfer to our hospital for pacemaker implantation.

The patient’s medical history included hypertension and a left renal artery stent implantation in 2009, addressing left renal artery stenosis. There was no history of diabetes. Upon physical examination, her height was 155 cm, weight was 50 kg, and inspection of the chest wall revealed scars and wrinkles on the right subclavian skin (see Supplementary material online, *Figure S1*). Percussion indicated a normal cardiac boundary, and there was no peripheral oedema.

**Figure 2:**
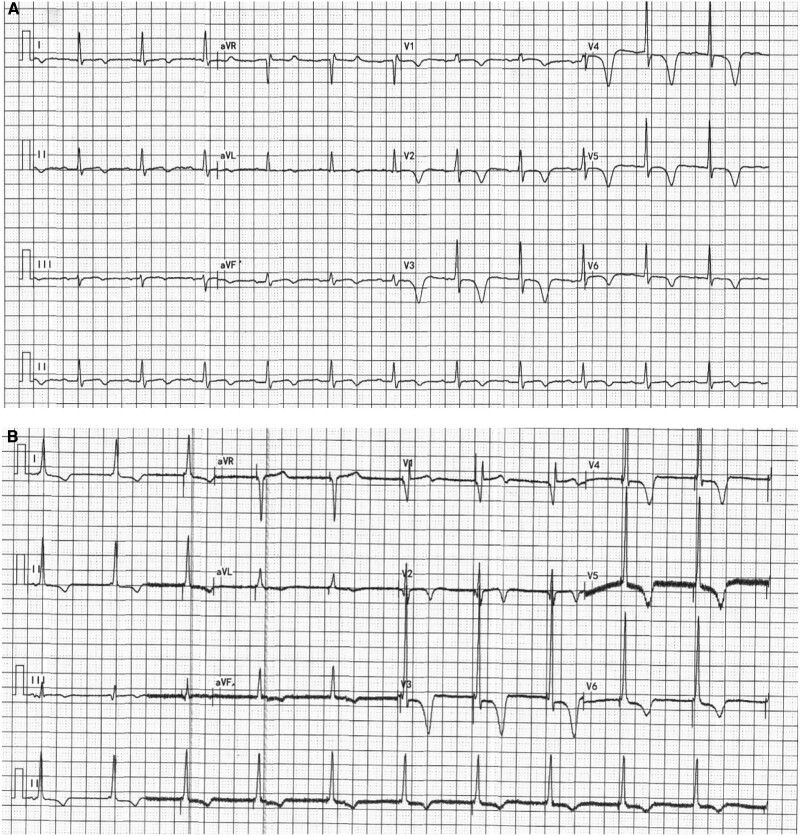
(*A*) The pre-operative electrocardiogram displayed sinus rhythm along with ST-segment depression and T-wave inversion in leads V1–V6. (*B*) The post-operative electrocardiograms revealed a QRS duration of 110 ms within the normal range.

The laboratory tests indicated normal liver and kidney function, absence of anaemia, and an NT-proBNP level of 1807 pg/mL. The electrocardiogram (ECG) displayed sinus rhythm, along with QRS duration 110 ms, ST-segment depression, and T-wave inversion in leads V1–V6 (*Figure 2A*). Echocardiography revealed mild tricuspid regurgitation, each cardiac chamber within normal range, and reduced anterior wall contraction activity, resulting in a left ventricular ejection fraction of 50%.

Considering the clinical history, symptom presentation, and timing, the syncope was attributed to pre-automatic pause. Given the patient’s age and low pacing rate, a leadless pacemaker was recommended. However, the leadless pacemaker is much more expensive than the single-chamber pacemaker in our country. The patient could not afford the leadless pacemaker and opted for a cost-effective single-chamber pacemaker. Consequently, we chose LBBAP, considering the physiological pacing strategy and the patient’s economic capability.

**Figure 3:**
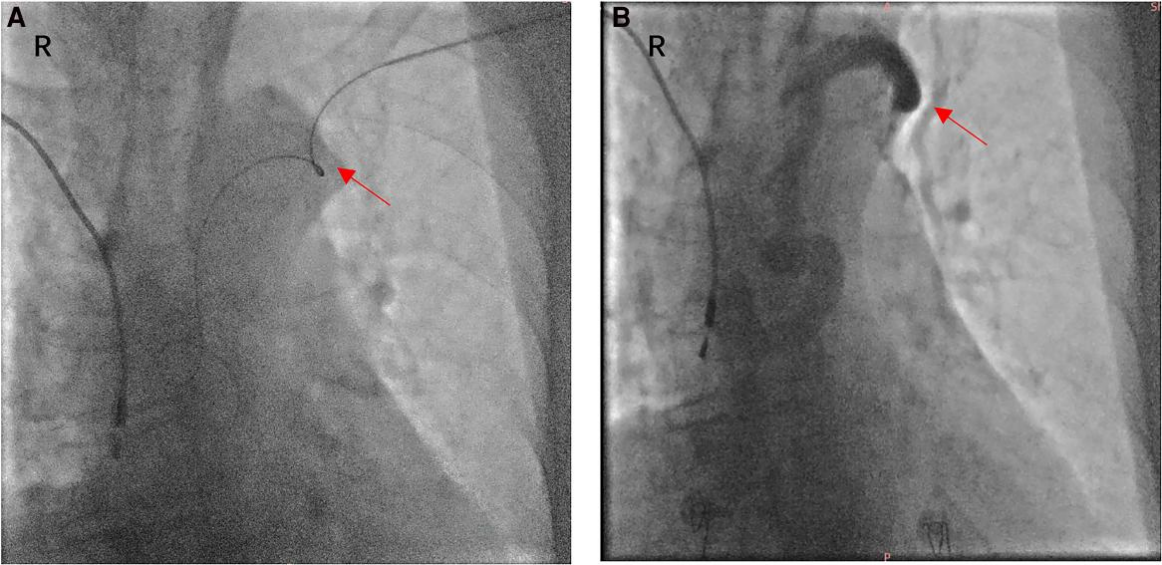
(*A*) The guide wire failed to reach the superior vena cava after puncturing left subclavian vein. (*B*) Venography showed that the left brachiocephalic vein was occluded (the arrows).

Due to the patient’s past right subclavian pocket infection, the presence of scars, and residual leads, local anaesthesia was administered for a left subclavian incision. However, during the procedure, the guide wire failed to reach the superior vena cava (*Figure 3A*), revealing occlusion in the left brachiocephalic vein through venography (*Figure 3B*). After discussing operational risks and treatment alternatives with the patient, we modified the plan and proposed implantation via the right iliac approach, which the patient accepted.

**Figure 4:**
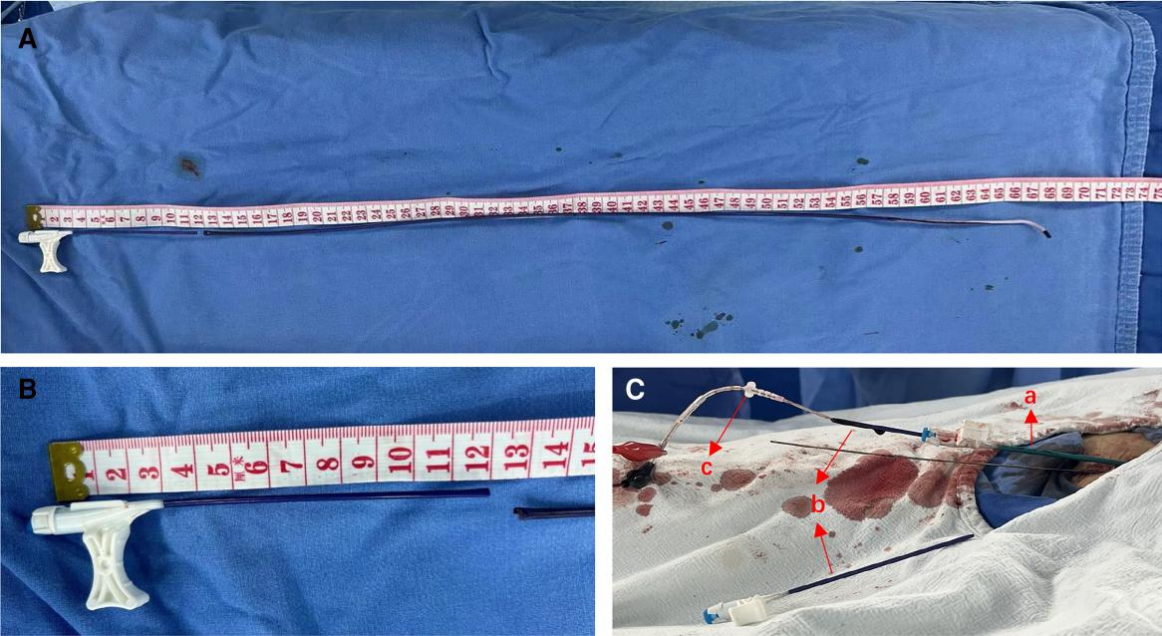
(*A*) Medtronic Attain Select II 6248V-130p was used as the inner sheath. (*B*) The haemostatic valve and 12 cm of the proximal end of the inner sheath was cut for increased usable lead length. (*C*) Medtronic Attain Command 6250V-MB2X as the outer sheath (a); Medtronic Attain Select II 6248V-130p as the inner sheath (b); and Medtronic 3830-69 cm as the pacing lead (c).

**Figure 5:**
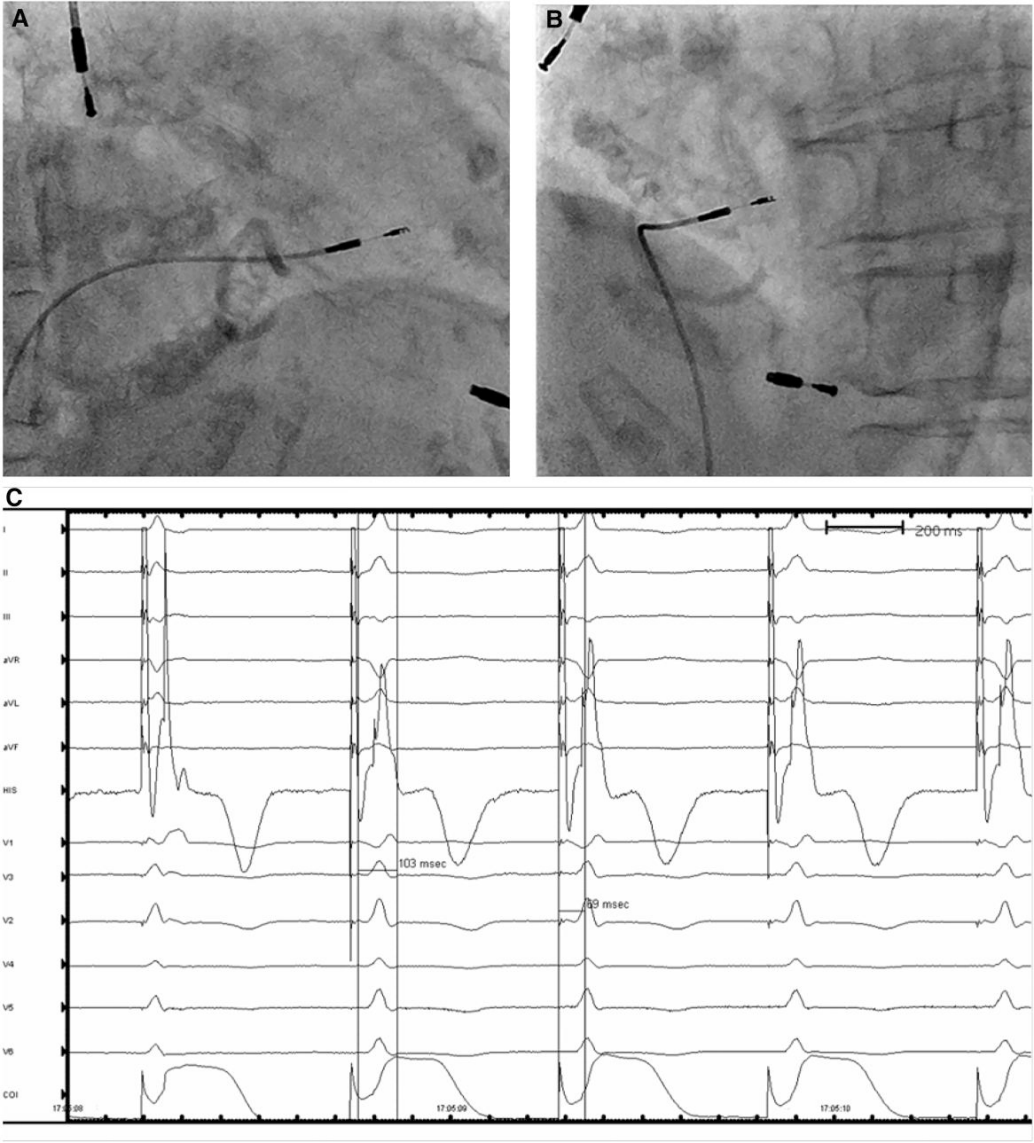
The left bundle branch area pacing lead was screwed into the right ventricular septum (*A*) under the projection of right anterior oblique 30 view and (*B*) under the projection of left anterior oblique 30 view. (*C*) Left bundle branch area pacing threshold testing exhibited an incomplete right bundle branch block pattern in V1, with a width of 103 ms and an left ventricular activation time of 69 ms in V6 at a low output.

After successfully puncturing the right iliac vein guided by fluoroscopy instead of ultrasound, we inserted the guide wire. The initial sheath used (Medtronic C315His) was directed towards the right ventricular free wall consistently. Subsequently, we switched to a left ventricular sheath (Medtronic Attain Command 6250V-MB2X), and the pacing lead (Medtronic 3830-69 cm) was introduced into the right ventricle through this sheath. However, identifying a suitable site for fixing the distal end of the lead perpendicularly to the interventricular septum proved challenging. Attempting Medtronic Attain Select II 6248V-130p as the inner sheath, we guided the outer sheath (Medtronic Attain Command 6250V-MB2X) into the right ventricle. The inner sheath was then positioned anterior and inferior to the His bundle, perpendicular to the ventricular septum. The 3830 lead was inserted along the inner sheath, and for increased usable lead length, the haemostatic valve was removed, and 12 cm of the proximal end of the inner sheath was cut (*Figure 4A–C*). The lead was screwed into the right ventricular septum until the unipolar QRS complex morphology exhibited an incomplete right bundle branch block pattern in V1, with a width of 103 ms and an left ventricular activation time of 69 ms in V6 at a low output (*Figure 5A–C*). The measurement of V6–V1 interpeak interval was 35 ms (see Supplementary material online, *Figure S3*). The pacing threshold was 1.0 V at 0.4 ms, sensing was 11 mV, and impedance was 880 Ω; in bipolar mode, the threshold was 5 V. The sheaths were split, and the lead was secured near the puncture site. The generator (Xintong LD300SR) was implanted in the lower-right abdominal pocket (see Supplementary material online, *Figure S2*). The post-operative ECG revealed a QRS duration of 110 ms within the normal range (*Figure 2B*).

During the 6-month follow-up visit after the pacemaker implantation, the patient exhibited mobility without lower limb symptoms or oedema. The pacing system continued to function properly, and the ventricular pacing per cent was 36.8% (see Supplementary material online, *Figure S4*).

## Discussion

Left bundle branch area pacing presents itself as a promising alternative to the conventional right ventricular pacing, and this case marks the first instance, to our knowledge, utilizing an iliac approach. Although iliac or femoral cardiac implantable electronic device implantation cases have been previously described,^8–11^ such approaches have been infrequently employed due to the availability of surgical and leadless pacemaker alternatives.

Right ventricular pacing, a longstanding strategy with over 60 years of clinical use, has demonstrated utility. However, when applied at the endomyocardium rather than the conduction system, it is associated with an elevated risk of atrial fibrillation, heart failure, and mortality, particularly in ventricular pacing-dependent patients.^12^ The His bundle pacing (HBP) method, deemed the most physiological pacing strategy, has shown a reduced risk of heart failure hospitalization and mortality.^12^ Nevertheless, the higher pacing threshold and lower R-wave amplitude of HBP have limited its routine clinical application due to safety concerns.^2,3^ Recently, LBBAP has emerged as a novel physiological pacing strategy, providing an alternative to HBP. By pacing at a more distal and deeper area than HBP, capturing the left bundle branch (LBB), including its trunk and proximal fascicles, is relatively straightforward given the wide network of the LBB.^4^ As demonstrated in various observational studies, LBBAP exhibits a low and stable threshold, along with a high R-wave amplitude.^5–7^ In this particular case, the patient’s history of myocardial infarction led to the recommendation of LBBAP to prevent further deterioration of cardiac function. Considering the patient’s low pacing rate and cost considerations, the decision was made to implant a single-chamber pacemaker with LBBAP.

The success of our procedure hinges on the efficiency of the delivery catheter system. Standard His catheters and left ventricular sheaths pose challenges in achieving a perpendicular orientation to the ventricular septum. The Attain Select II + SureValve delivery catheter system (Medtronic Attain Select II 6248V-130p) proves advantageous for positioning the 3830 lead. Its distal end features a 130° bend, and its inner diameter accommodates the 3830 lead comfortably. Notably, the length of the inner catheter exceeds that of the 3830 lead (69 cm), necessitating the trimming of the proximal end of the outer sheath. While cutting the haemostatic valve at the sheath’s proximal end may lead to excessive blood loss, the minimal difference in inner diameter between Medtronic Attain Select II 6248VI and the outer diameter of lead 3830 mitigates this concern. Dhakal *et al.*^10^ reported a femoral approach case for LBBAP without superior access, utilizing the Attain Select II delivery catheter system and modifying the inner sheath for lead insertion.

Comparing iliac access to femoral vein access, the advantage of iliac access lies in its minimal impact on lower limb activities, ensuring no disruption to patients’ daily lives and reducing the risk of lead dislocation. In a previous report, we discussed a case of Cardiac Resynchronization Therapy implantation via the iliac approach, detailing indications and puncture experiences.^11^ The key point of the external iliac vein puncture is at the 1–1.5 cm point inside the femoral artery pulsation above the inguinal fold, guided by fluoroscopy or ultrasound, but ultrasound guidance is unavailable at our centre. Tunnelling is not necessary. Some lead slack should be left to reduce the chance of dislodgement, and an anchoring suture is necessary to avoid the movement of leads. Subsequent follow-ups on the two patients in our centre affirmed the stability of leads implanted through the right iliac vein. Compared with the subclavian route, the risks of the iliac vein include iatrogenic retroperitoneal haematoma and the lead dislocation. However, the lead fracture is rare. Cefuroxime is prescribed as the prophylactic antibiotic treatment, which is the same as the subclavian access.

## Conclusion

It is the first described LBBAP implantation case through the iliac access, which affirms the feasibility and stability of LBBAP through iliac vein access when the superior venous route is inaccessible or contraindicated. Significantly, this approach presents itself as a practical alternative, mitigating unnecessary risks associated with thoracotomy and epicardial lead placement.

## Supplementary Material

ytae486_Supplementary_Data

## Data Availability

The data supporting this article will be made available upon reasonable request to the corresponding author.

## Lead author biography

Dr. Xu is from Shanghai Zhongshan Hospital, Fudan University. She has been a cardiologist for over 10 years, hoping to exchange the clinical experience with colleagues all over the world.

## Supplementary material

**Consent:** The patient provided written informed consent for the publication of this article in accordance with COPE guidelines.

**Funding:** This study was funded by the grant (82070242) to L.X. from the Surface Project of National Natural Science Foundation of China.
