# Supplementary material for: Left bundle branch area pacing from the iliac approach in a patient without superior access: a case report
Source: Eur Heart J Case Rep. 2025 Feb 11;9(2):ytae486. doi: 10.1093/ehjcr/ytae486 (PMC11811417; doi:10.1093/ehjcr/ytae486)
Supplement: ytae486_Supplementary_Data [file ytae486_supplementary_data.docx]

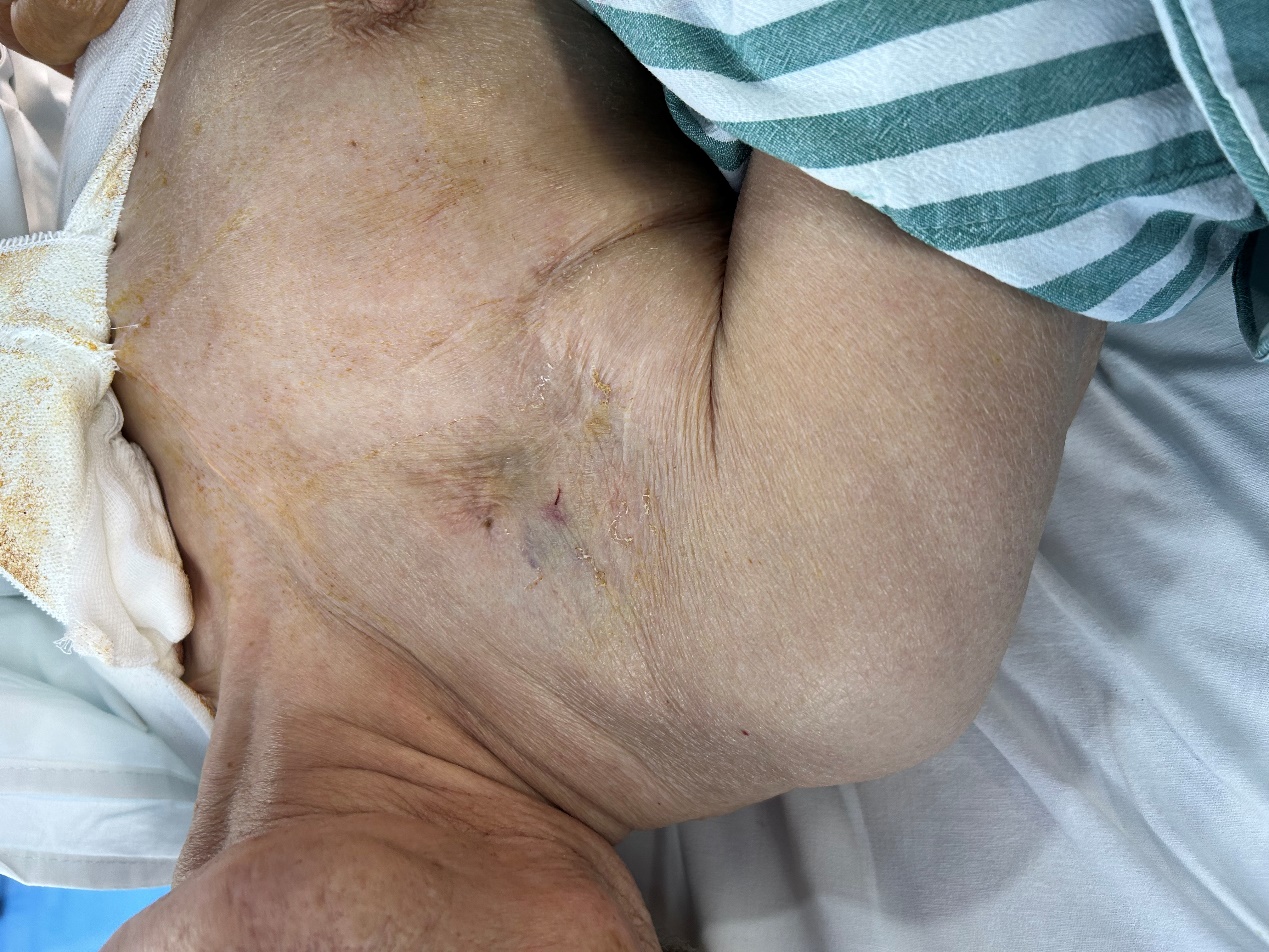


Supplementary Figure 1 The inspection of the chest wall revealed scar and wrinkles on the right subclavian skin of the patient.


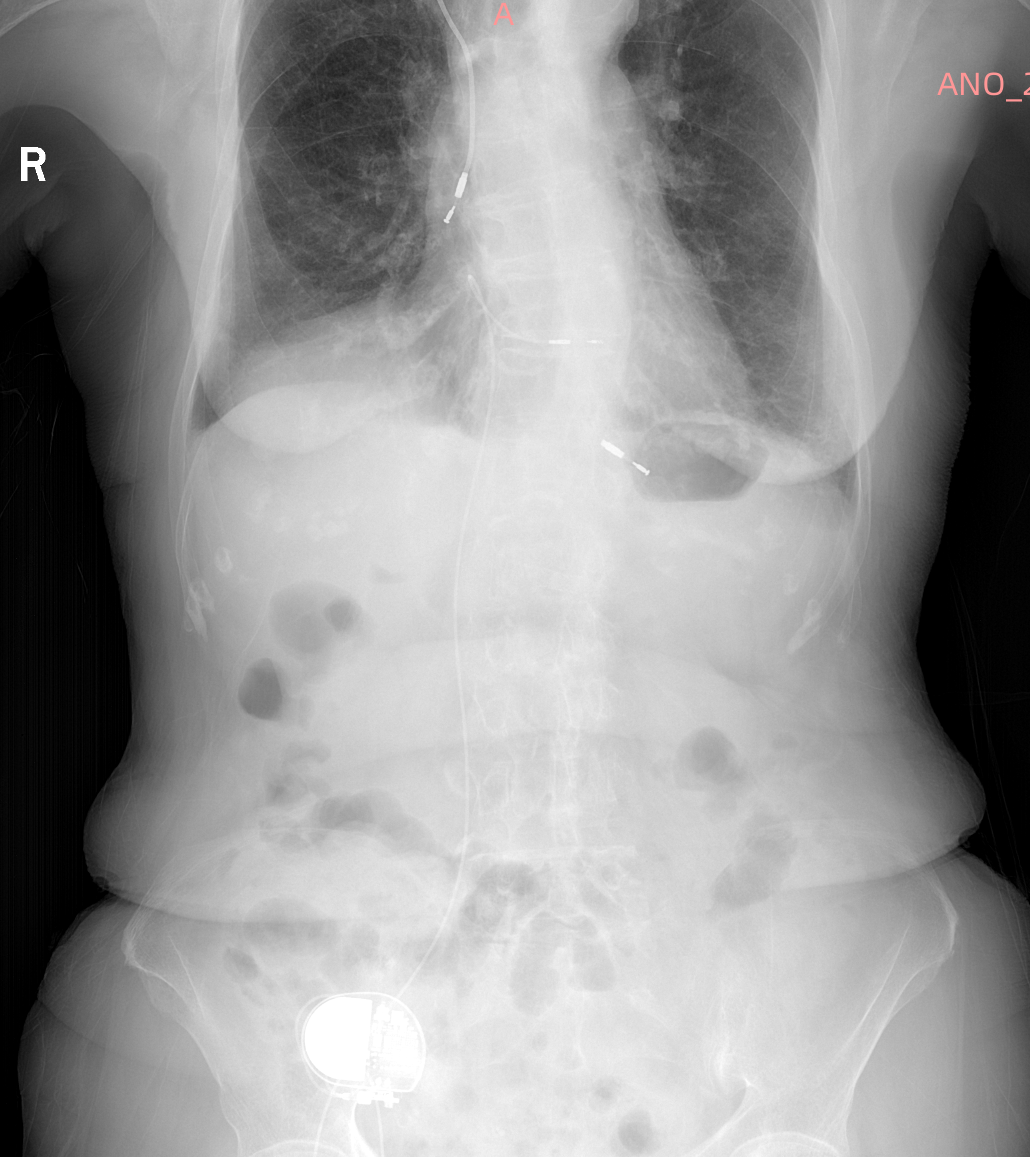


Supplementary Figure 2 The chest, abdominal, and pelvic radiograph shows the final lead and generator positions.





Supplementary Figure 3 The measurement of V6-V1 interpeak interval was 35ms.


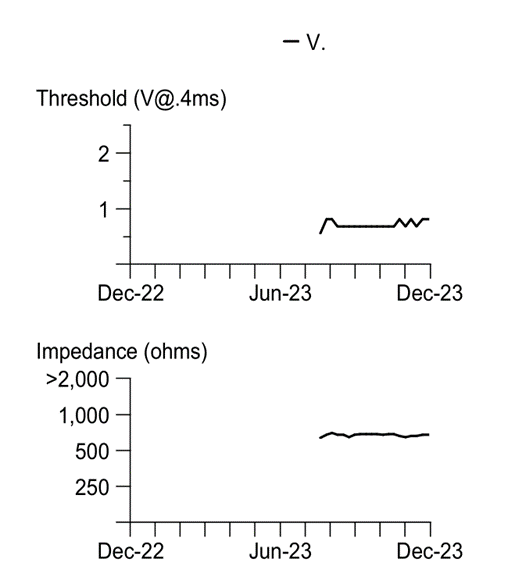

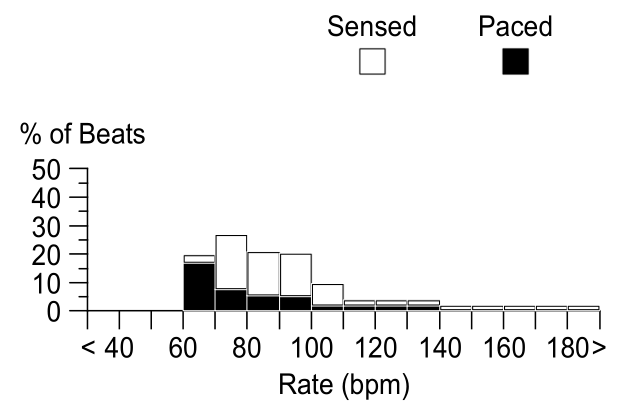

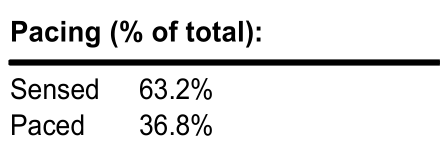


Supplementary Figure 4 Stable parameters observed six months post-operatively.
